# Supplementary material for: An Ionic-Liquid-Imprinted Nanocomposite Adsorbent: Simulation, Kinetics and Thermodynamic Studies of Triclosan Endocrine Disturbing Water Contaminant Removal
Source: Molecules. 2022 Aug 23;27(17):5358. doi: 10.3390/molecules27175358 (PMC9457669; doi:10.3390/molecules27175358)
Supplement: Supplementary file 1 [file molecules-27-05358-s001.zip › molecules-1842638-supplementary.pdf]

Supplementary Information

An Ionic-Liquid-Imprinted Nanocomposite Adsorbent: Simulation, Kinetics and Thermodynamic Studies of Triclosan Endocrine Disturbing Water Contaminant Removal

Imran Ali [1,2,*], Gunel T. Imanova [3], Hassan M. Albishri [2], Wael Hamad Alshitari [4], Marcello Locatelli [5], Mohammad Nahid Siddiqui [6] and Ahmed M. Hameed [7]

1 Department of Chemistry, Jamia Millia Islamia (Central University), New Delhi 110025, India
2 Department of Chemistry, King Abdulaziz University, Jeddah 21589, Saudi Arabia
3 Department of Physical, Mathematical and Technical Sciences, Institute of Radiation Problems, Azerbaijan National Academy of Sciences, AZ 1143 Baku, Azerbaijan
4 Department of Chemistry, College of Science, University of Jeddah, Jeddah 21589, Saudi Arabia
5 Department of Pharmacy, University "G. d'Annunzio" of Chieti-Pescara, Build B, Level 2, Via dei Vestini, 31, 66100 Chieti, Italy
6 Department of Chemistry and IRC Membranes and Water Security, King Fahd University of Petroleum and Minerals (KFUPM), Dhahran 31261, Saudi Arabia
7 Department of Chemistry, Faculty of Applied Sciences, Umm Al-Qura University, Makkah 21955, Saudi Arabia
* Correspondence: drimran.chiral@gmail.com or drimran_ali@yahoo.com

1. Experimental

1.1 Chemicals and reagents

The used triclosan was purchased from Sigma-Aldrich Chemical Co. USA. Copper acetate, formic acid of AR grade and methanol of HPLC grade were supplied by Fisher Scientific, Fairlawn, New Jersey, USA. The solutions of triclosan (1.0 mg/L) was prepared in ethanol for HPLC studies while the dilution was done with water for adsorption studies.

1.2 Instruments

FT-IR used was Nicolet 400 spectrometer. The scanning electron microscope was of Aztec X-Max energy dispersion spectrometer, Oxford Instruments. The transmission electron microscopy (TEM) used was of JEM 2010 instrument JEOL, USA. Triclosan analysis was carried out on the HPLC system of Shimadzu (Osaka, Japan) with Sunniest RP Aqua C_{28} (25 cm x 4.6 mm id) of Chromanik, Japan. The other instruments used were pH meter, centrifuge, thermostat water bath.

1.3 Simulation study

1.3.1 Preparation of triclosan and *N*-methyl butyl imidazolium bromide pdb files

Triclosan (receptor) and N-methyl butyl imidazolium bromide (ligand) structures were prepared in Marvin Sketch and saved in pdb files. After that, the pdb files were accessed in AutoDock Tools (ADT) 4.2 to add the non-polar hydrogen atoms, followed by Gasteiger charger assigned the molecules. After adding all the materials required for the docking study, the pdb file of N-methyl butyl imidazolium bromide ionic liquid was saved as pdbqt format and used as ligand. Both pdb files of triclosan were converted into pdbqt format using AutoDock Tools (ADT) 4.2 one by one, and docking was achieved by using ADT considering all the rotatable bonds of the ligand as rotatable, and the receptor as rigid. The grid box size of 60 × 80 × 110 A° with 0.375 A° spacing was used.

1.3.2 Docking Methods

All the pdbqt formatted files were docked one by one using AutoDock vina program. Before the docking method using AutoDock vina, all the coordinates were set (x = 30.054, y = 22.75, and z = 4.171). Many independent docking runs were applied for the lowest free energy of binding confirmation from the largest cluster.

1.3.3 Docking Analysis

The analysis of the modes of the interactions and number of hydrogen bonds were studied by PyMOL. On the other hand, hydrophobic interactions between triclosan and *N*-methyl butyl imidazolium bromide were also studied using LigPlot 1.4.5.

2. Adsorption study

2.1 Modeling

The experimental data were analyzed by using following models.

Table S1. Langmuir, Freundlich, Temkin, Dubinin–Radushkevich adsorption models*.

Langmuir	Freundlich	Temkin	Dubinin–Radushkevich
Linear forms of the equations			
$\frac{1}{Q_e}=\frac{1}{Q_{\max}}+\frac{1}{Q_{\max}*b}*\frac{1}{C}$	$\lg Q_e=\frac{1}{n}*\lg C+\lg k$	$Q_e=B*\ln K_T+B*\ln C$	$\ln Q_e=\ln Q_{\max}-K_{ad}*\varepsilon^2$
Non-Linear forms of the equations			
$Q_e=Q_{\max}*\frac{b*c}{1+(b*c)}$	$Q_e=k*C^{1/n}$	$Q_e=B*\ln(K_T*C)$ (9)	$Q_e=Q_{\max}*\exp(-K_{ad}*\varepsilon^2)$

Q_{max} - maximum adsorption capacity, mg g^{-1}; *C* - equilibrium concentration, mg L^{-1}, *b* - adsorbent-adsorbate affinity distribution (adsorption energy), L mg^{-1}; *n* - adsorption favorability indicator; *k* - adsorption capacity of the adsorbent, mg g^{-1} L mg^{-1}; K_T - equilibrium binding constant corresponding to the maximum binding energy, L mg^{-1}; B - constant related to the heat of adsorption; K_{ad} – Dubinin–Radushkevich isotherm constant, mol^2 $k^{-1}J^{-2}$; ε - Polanyi potential, kJ mol^{-1}.

Table S2. Models implemented to describe the adsorption kinetics and mecahnism*.

Model	Equation
Pseudo-first-order	$\lg(Q_e-Q_t)=\lg(Q_e)-\frac{k_1*t}{2,303}$
Pseudo-second-order	$\frac{t}{Q_t}=\frac{1}{k_2*Q_e^2}+\frac{1}{Q_e}*t$
Elovich	$Q_t=\frac{1}{\beta}*\ln(\alpha*\beta)+\frac{1}{\beta}*\ln(t)$
Intraparticle diffusion	$Q_t=k_{id}*t^{0,5}+c$
Liquid film diffusion kinetic	$-ln(1-F)=k_{fd}*t$

* *t* – adsorption time, min; Q_e – adsorption at equilibrium, mg g^{-1}; Q_t – adsorption at time *t*, mg g^{-1}; k_1 – pseudo-first-order adsorption rate constant (min^{-1}); k_2 – pseudo-second-order adsorption rate constant, g mg^{-1} min^{-1}; k_{id} – intraparticle diffusion coefficient, min $^{-1/2}$; *c* – boundary layer thickness constant, mg g^{-1}; $F=Q_t/Q_e$ – fractional attainment of equilibrium; k_{fd} – adsorption rate constant, sm s^{-1}; α – initial flow rate, min^{-1} mg g^{-1}; β - desorption constant (degree of surface coverage and activation energy of chemisorptions), g mg^{-1}.

2.2 Thermodynamics calculations

$\Delta G^0 = -RT \ln(K^0_e)$ ---1

$\Delta G^0 = \Delta H^0 - T\Delta S^0$ ---2

Combine equation 1 and 2 then

$\ln(K^0_e)$ = [$-H^0/R$]. [$1/T$]. [$\Delta S^0/R$] ---3 or $Ln(K_e^0) = \frac{-\Delta H^0}{R} . \frac{1}{T} + \frac{\Delta S^0}{R}$

By constructing a plot of *ln(Ke°) versus 1/T*, from the intercept is calculated the change in entropy (ΔS°) and by the slope, it is possible to calculate the change in enthalpy (ΔH°).

The correct way to calculate the equilibrium constant for adsorption system is to obtain isotherms of adsorption at different temperatures and making the nonlinear fitting of the isotherms. From the best-fitted model at the different temperatures, the equilibrium constant is obtained one for each isotherm at a given temperature. This equilibrium constant obtained in the isotherms (usually expressed in L mg^{-1}) must become dimensionless for being applied in the Vant´Hoff equation. In this sense, it is recommended to use Eq 4 below described for calculating the thermodynamic parameters by the Vant´Hoff equation (Eq. 3).

$$K^0_e = \frac{(1000.K_g.\text{molecular weight of adsorbate}).[\text{Adsorbate}]^\circ}{\gamma} \quad \text{------4}$$

Kg = Langmuir constant (L/mg), $[\text{Adsorbate}]^0$ = mol/L and γ = coefficient of activity and is equal to 1 in case of adsorption experiments as these are at very dilute conc. In this way, calculate K^0_e and draw a plot of *Ln(Ke°) versus 1/T*, from the intercept is calculated the change in entropy (ΔS°) and by the slope change in enthalpy (ΔH°) is calculated.

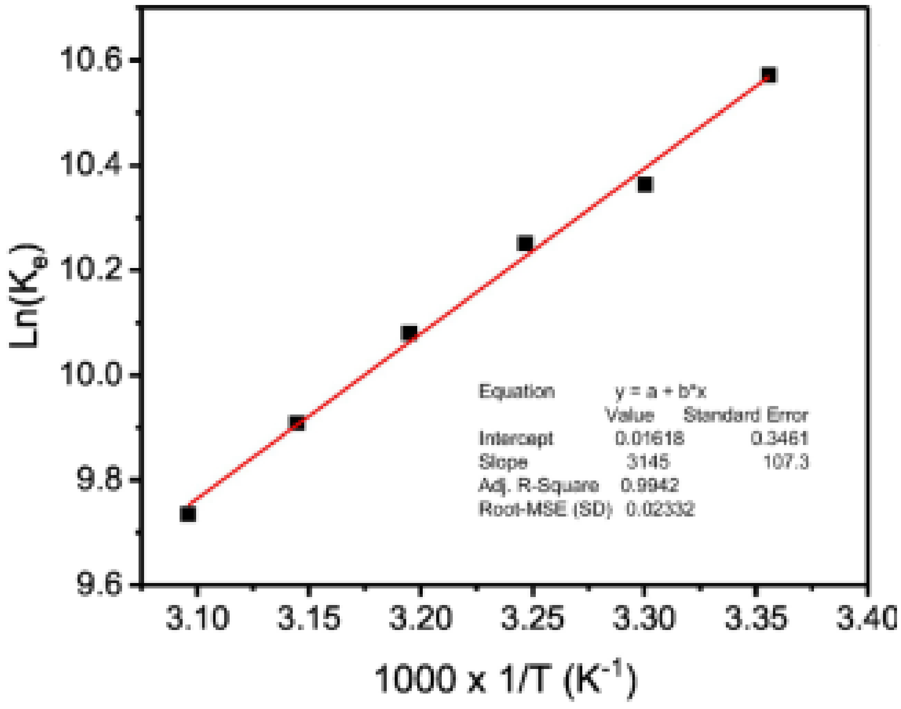

The values of free energy were calculated by the following equation.

ΔG^0 = -RT lnKc [Kc is Langmuir constant b (L/mg)].

b is not dimension less and as per IUPAC it should be dimensionless. For this use following equation for conversion was used. In our case 'consider the exchange adsorption'.

$K^o = b \times M_{Tricolsan} \times 10^6 \times 55.4$; where M is the molar mass.
